# Unveiling subcellular secrets: A novel sensor to visualize heme distribution in plants

**DOI:** 10.1093/plphys/kiae388

**Published:** 2024-07-23

**Authors:** Prateek Jain

**Affiliations:** Plant Physiology, American Society of Plant Biologists; Department of Biology, The University of North Carolina at Chapel Hill, Chapel Hill, NC 27599-3280, USA

Heme is just not a magic ingredient for the “Impossible burger” that gives the burger its meaty flavor but an essential molecule for every biological function in living organisms. It is a vital cofactor and signaling molecule that acts as a circlet of atoms to hold iron like a diamond ring. In contrast to animals, where heme is synthesized in mitochondria, in plants heme synthesis takes place in plastids, followed by its distribution to other cellular compartments (Wang et al. 2022). Inside cells, heme is usually present as “labile (loosely bound),” “regulatory (signaling),” “free (non-protein bound),” or “heme pool” (Gallio et al. 2021). Owing to the importance in cellular signaling, several methods, including biochemical and quantitative assays, have been developed to determine the subcellular distribution of heme (Richter et al. 2019). However, quantification of intracellular heme in plants is more complex due to the intricate purification and excess of tetrapyrrole end-products (Espinas et al. 2012). Considering the importance of heme in biological processes, effective measurements of heme distribution in living cells are needed.

In this issue of *Plant Physiology*, Wen and Grimm present a new method for assessing free heme distribution in subcellular compartments in plants (Wen and Grimm 2024). The study is an extension of earlier work by Hanna et al. (2016), in which a novel heme sensor, HS1, and its variant HS1(M7A) were developed and used to determine the subcellular distribution of free heme in yeast (Hanna et al. 2016). HS1 is a tripartite fusion protein with cytochrome b562 (heme binder), far-red fluorescent protein mKATE2 (fluorescence after heme binding), and EGFP protein (fluorescence independent of heme) (Fig. A) (Hanna et al. 2016). The 3 different components help HS1 to ratiometrically image free heme and to quantify total heme in subcellular compartments. To visualize heme distribution in different organelles, the authors tagged HS1 and its variant HS1 (M7A) with signaling peptides for plastid (RbcS), mitochondria (COX4), and nucleus (SV40) and transiently expressed them in tobacco leaves. By comparing the relative fluorescence ratios within and between the low- and high-affinity sensors, the authors conclude that the heme level is lowest in the nucleus and slightly higher in the chloroplasts and mitochondria relative to the cytoplasm.

**Figure.**
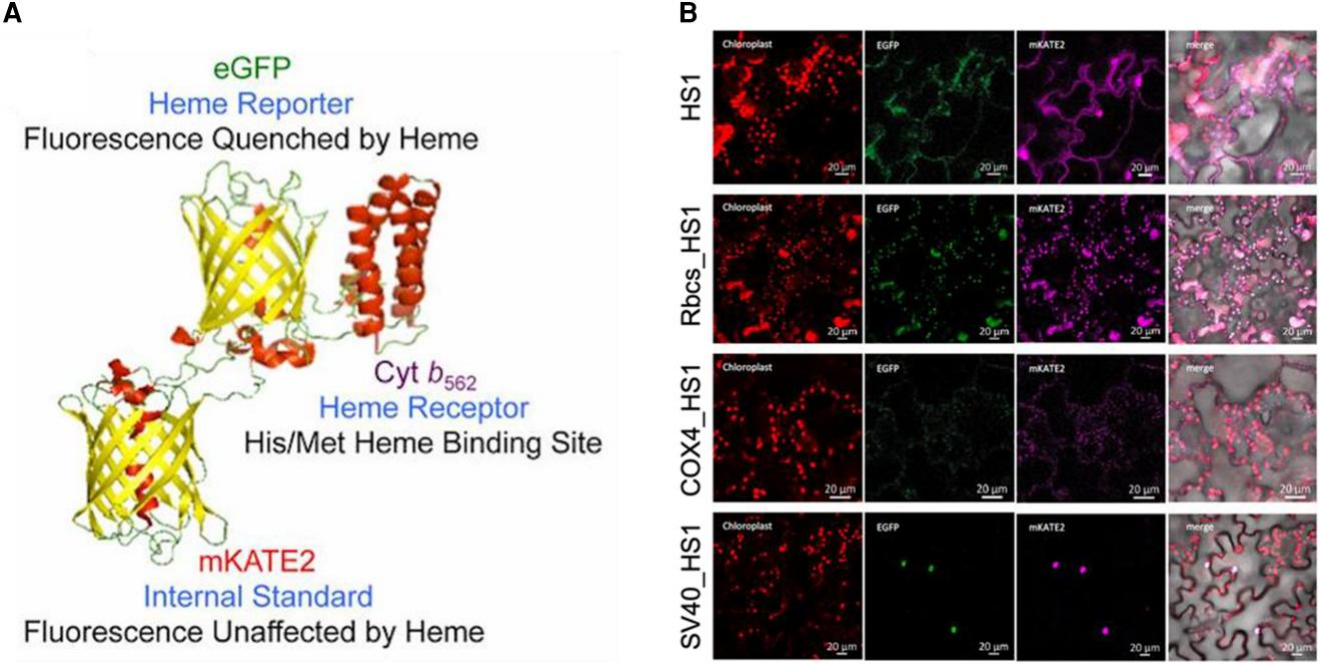
The heme sensor HS1 can visualize intracellular free heme. **A)** Structural modules of HS1 (figure adapted from Hanna et al. 2016). **B)** The fluorescence signals of HS1 in different cellular compartments of transiently transformed leaf. HS1 was fused with localization peptides for plastid (RbcS), mitochondria (COX4), and nucleus (SV40) to detect the available free heme.

That HS1 is a non-invasive tool for determining subcellular distribution of free heme motivated the authors to locate it intracellularly in plants. To do so, the authors treated HS1 transformed tobacco leaf sections with different concentrations of 5-aminolevulinic acid (ALA), a precursor for heme synthesis, and with gabaculine, an inhibitor of ALA synthesis pathway (Wen and Grimm 2024). ALA treatments caused lower EGFP/mKATE2 fluorescence signals in chloroplast and mitochondria due to the higher heme levels, while nucleus, which has a low heme content, shows no change in EGFP/mKATE2 fluorescence signals post ALA and gabaculine treatments.

Having confirmed that chloroplast and mitochondria are sources of heme, the authors then studied the relationship between leaf age and heme synthesis. The authors selected tobacco leaves of different developmental stages and transiently transfected them with the heme sensor. Wen and Grimm (2024) observed a lower EGFP/mKATE ratio in chloroplast of young leaves due to higher free heme and elevated tetrapyrrole biosynthesis. However, the nucleus and mitochondria of older and new leaves have similar fluorescence ratios, confirming the subcellular differences in heme distribution.

Finally, to assess heme distribution in planta, the authors developed transgenic plants of Arabidopsis and tobacco expressing HS1. Ectopic expression of heme sensor does not cause detrimental effects in *Arabidopsis*. The differential expression of HS1 also had no influence on the ratio of EGFP to mKATE2 fluorescence and is directly proportional to available free heme (Fig. B). Furthermore, ALA treatments caused lower EGFP:mKATE2 fluorescence signals, which indicate higher heme content, in plastid and mitochondria compared with nucleus, where heme is stable.

This finding establishes the role of HS1 in monitoring the heme content subcellularly in plants. To visualize the intracellular heme, Wen and Grimm (2024) tagged HS1 and its variant HS1(M7A) with signal peptides that send HSs to plastids or chloroplasts. The authors also showed that young leaves produce more heme than older leaves. In conclusion, HS1 provides a nondestructive mode for determining the intracellular heme content in plants.
